# Calibration and measurement control based on Bayes statistics

**DOI:** 10.1155/S1463924689000325

**Published:** 1989

**Authors:** Katalin M. Hangos, László Leisztner, Miroslav Kárný

**Affiliations:** 1 Computer and Automation Institute Hungarian Academy of Sciences PO Box 63 Budapest H1502 Hungary; 2 Institute of Forensic Science PO Box 314/4 Budapest H1903 Hungary; 3 Institute of Information Theory and Automation Czechoslovak Academy of Sciences Pod vodárenskou vezi 4 Praha 8 18208 Czech Republic

## Abstract

The Bayesian methodology described in this paper has the inherent capability of choosing, from calibration-type curves, candidates which are plausible with respect to measured data, expert knowledge and theoretical models (including the nature of the measurement errors). The basic steps of Bayesian calibration are reviewed and possible applications of the results are described in this paper. A calibration related to head-space gas chromatographic data is used as an example of the proposed method. The linear calibration case has been treated with a log-normal distributed measurement error. Such a treatment of noise stresses the importance of modelling the random constituents of any problem.


Journal of Automatic Chemistry Vol. 11, No. 4 (July–August 1989), pp. 149–155

### Calibration and measurement control based on Bayes statistics

**Katalin M. Hangos**

*Computer and Automation Institute, Hungarian Academy of Sciences, H1502 Budapest, PO Box 63, Hungary*

**László Leisztner**

*Institute of Forensic Science, H1903 Budapest, PO Box 314/4, Hungary*

**and Miroslav Kárný**

*Institute of Information Theory and Automation Czechoslovak Academy of Sciences, 18208 Praha 8, Pod vodárenskou vezi 4, Czechoslovakia*

*The Bayesian methodology described in this paper has the inherent capability of choosing, from calibration-type curves, candidates which are plausible with respect to measured data, expert knowledge and theoretical models (including the nature of the measurement errors). The basic steps of Bayesian calibration are reviewed and possible applications of the results are described in this paper. A calibration related to head-space gas chromatographic data is used as an example of the proposed method. The linear calibration case has been treated with a log-normal distributed measurement error. Such a treatment of noise stresses the importance of modelling the random constituents of any problem.*

### Introduction

The large number of papers dealing with problems of calibration (for example [1, 2 and 10]) shows the importance of calibration to any method of analytical measurement. The published literature addresses a variety of aspects of this complex and so far unsolved problem.

The Bayes methodology applied in this paper proved to be an efficient tool for handling problems which are similar to those found in calibration. The method allows candidates which are plausible with respect to measured data, expert experience and theoretical models (including the nature of the measurement errors) to be highlighted in calibration curves. The potential of the Bayesian framework was recognized by the editorial board of the journal *Technometrics,* who started a discussion about its relevance to the calibration problem [3]. The discussion was, however, devoted mainly to the theoretical aspects of Bayesian calibration and only linear calibration with normally distributed measurement error was considered in depth.

The authors attempt to show here how the Bayesian approach can be used and to illustrate that it is a *practical* and powerful tool for solving calibration problems (see also [11]). This paper is complementary to Kárný and Hangos (1989, [9]).

### The Bayesian view of the calibration problem

The problem of quantitative measurement can be described with a set of measured values

$$Y = \{y_i, i = 1, \ldots, N\} \quad (1)$$

which corresponds to a given set of samples with concentrations

$$X = \{x_i, i = 1, \ldots, N\}. \quad (2)$$

The calibration aims to estimate the mutual relation of measurements $Y$ and concentrations $X$ by evaluating measurements $Y_c$ for known values of the so-called *control* samples $X_c$.

Calibration is usually understood to be the fitting of a calibration curve of a given form $y = F(x,\theta)$, parametrized by a multivariate parameter $\theta$ to the data pairs $(Y_c,X_c) = \{(y_{ic}, x_{ic}), i = 1, \ldots, N_c\}$. A point estimate $\hat{\theta}$ of $\theta$ is then taken as the calibration result, i.e. measurements and (unknown) samples are supposed to be related by $y = F(x, \hat{\theta})$.

The following drawbacks of such a calibration can be serious in difficult calibrations. (1) The method of fitting is often not adapted to the character of the measurement errors (for example least-squares estimation behaves poorly in the presence of outliers); (2) The information about the precision of the calibration is difficult to obtain (especially where few control samples are available); (3) The information content of the calibration data is often substantially reduced due to generic non-linearity of the performed data reduction. Thus the random measurement errors are transformed into a systematic error, cf. the analysis in reference [4].

The Bayesian methodology relies on the assumption that random and uncertain quantities have a probabilistic structure: they can be described by tools of probability theory. The relationship of measurements $Y$ and concentrations $X$ is expressed in the form of mutual probability density function (the term 'probability density funcion' will be abbreviated to p.d.f.) $p(Y,X)$. A calibration is needed when this description is not completely known and when there is uncertainty about the value of a multivariate parameter $\beta$. Within the Bayesian framework it means that p.d.f. $p(Y,X,\beta)$ is specified. Using the elementary chain rule for p.d.f.s [6] this joint p.d.f. can be expressed in a form which is closer to the standard formulation

$$p(Y,X,\beta) = p(Y|X,\beta)p(X|\beta)p(\beta) = p(Y,X|\beta)p(\beta). \quad (3)$$

The conditional probability density function (c.p.d.f.) $p(Y|X,\beta)$ is a probabilistic counterpart of the parametrized calibration curve $F$ for whole set of data pairs $\{y_i, x_i\}$; the c.p.d.f. $p(X|\beta)$ describes the 'population' of measured samples and $p(\beta)$ expresses available knowledge (based on expert experience or previous data) about $\beta$. The required p.d.f. relating concentrations and

0142–0453/89 $3.00 © 1989 Taylor & Francis Ltd.

measurements $p(Y,X)$ is found from the p.d.f. (3) as follows [6]

$$p(Y,X) = \int p(Y,X,\beta)\mathrm{d}\beta. \quad (4)$$

The calibration data are used to modify (condition) this p.d.f. by the specific measured values $Y_c$ and $X_c$. Replacing particular quantities in equations (3) and (4) by their conditional versions gives the Bayesian solution of the calibration problem

$$p(Y,X,\beta|Y_c,X_c) = p(Y|X,\beta,Y_c,X_c)p(X|\beta,Y_c,X_c)p(\beta|Y_c,X_c) \quad (5)$$

$$p(Y,X|Y_c,X_c) = \int p(Y,X,\beta|Y_c,X_c)\mathrm{d}\beta. \quad (6)$$

Thus all the information about $\beta$ gained from the observed data $(Y_c,X_c)$ and from the expert knowledge $p(\beta)$ is contained in the p.d.f. of $\beta$ conditioned on the calibration data $p(\beta|Y_c,X_c)$, which can be simply derived from p.d.f. $p(Y_c,X_c,\beta)$ (see [7]). (In the remainder of this paper notation is often simplified by dropping the subscript c. The proper meaning will be clear from the context.)

The rather complex and general description above helps to combine all the available information (expert knowledge, theoretical models of deterministic relations, as well as of measurement errors and measured data) when solving different aspects of the calibration. The aim of this paper is to demonstrate how the principal term $p(Y,X|\beta)$ of the joint p.d.f. can be constructed and why it is generally necessary to extend parameters $\theta$ to $\beta$. The problems involved in quantifying in the expert knowledge by $p(\beta)$ is beyond the scope of this paper (for a discussion of this topic see [8]). The reader interested in further aspects of Bayesian statistics should consult [7].

It will help to start with a simple example.

*Example: The set and joint distribution of calibration parameters*

Let the *control* sample be non-random, i.e. there is neither a measurement nor a preparation error in it. The c.p.d.f. $p(X|\beta)$ is non-zero on the chosen values of concentrations $x = \{x_1, \ldots, x_N\}$, i.e.

$p(X|\beta) = \delta(x-X)$, where $\delta$ is the Dirac delta function.

Consequently, only $p(Y|X,\beta)$ has to be constructed. The theoretical calibration curve is assumed to be described by a deterministic function $F(x,\theta)$ and the measurement errors $\varepsilon_i$ of the particular measurements are independent and normally distributed with zero mean, i.e. only the random measurement error component is present. The variances of the random measurement errors $\varepsilon_i$ are concentration-dependent and this dependence is described by a known non-negative parametrized function of the form:

$\mathrm{var}(\varepsilon_i) = s(x_i,Q)R$, with $Q$ and $R$ unknown.

Under these assumptions the required c.p.d.f. is uniquely determined by the formulae

$$p(Y,X|\beta) = p(Y|X,\beta)\delta(x - X) \quad (7)$$

$$p(Y|X,\beta) = \prod_{i=1}^{N} [2\pi s(x_i,Q)R]^{-1/2} \cdot \exp\left[-\frac{(y_i - F(x_i,\theta))^2}{2s(x_i,Q)R}\right] \quad (8)$$

where parameter $\beta$ is the triple $(\theta,Q,R)$. Note that the conventional calibration curve is the (conditional) mean of the c.p.d.f. (8).

*Remarks*

(1) As the example shows, the parameters of the measurement error distribution enter into the set of calibration parameters. This supports the idea that conventional calibration results in information reduction: the neglected information about the character of the measurement error distribution is clearly relevant for optimum data handling. For instance, the detailed stochastic characteristics of the measured data which are lost due to the reduction are significant to any measurement diagnostics.

(2) If the measurement errors $\varepsilon_i$ also contain systematic error components (i.e. they have non-zero expectation), then the deviation of the conditional expectation of $y$ conditioned on $\beta$, taken as a function of $x$ from the theoretical calibration curve $F(x,\beta)$, is just this systematic error. The calibration can compensate for this difference if the measurement conditions, i.e. the properties of the measurement error, are kept constant during the calibration and measurement phases.

(3) Low dimensionality of the parameter $\beta$ is achieved by postulating the form (for example, Gaussian) of the measurement error distribution. This is, of course, restrictive. Including the discussed form in unknown quantities (so-called 'non-parametric estimation') would be preferable: it needs substantially more calibration measurements but provides more information about measurement errors.

(4) It is generally assumed that the measurement error has a Gaussian distribution. However, other distributions commonly found in practice [12] and other distributions will appear if there are any faults or failures in the measurement system or conditions.

(5) If it can be assumed that measurement errors are independent then the required c.p.d.f. modelling that measurement process has the following product form (this is known as a chain rule)

$$p(Y|X,\beta) = \prod_{i=1}^{N} p(y_i|x_i,\beta) \quad (9)$$

This case is often acceptable for correctly controlled analytical measurements so it is used for the rest of this paper. Also, concentrations and parameters are assumed to be independent, i.e. $\beta$ characterizes the measurement process only.

The general form of the chain rule [6] takes into account the fact that the result of a measurement can depend on the whole past measurement history. It has to be used when some dependency is encountered.

Clearly, there is a practical need to test whether the autocorrelated measurement errors are present both in the preliminary stages of analytical measurement and during routine measurements. A method for detecting this type of measurement error component is covered in reference [5].

(6) The generic term of the product (9) is

$$p(y|x,\beta) = \Gamma_y[F(x,\theta),s(x,Q)R]$$

where $\Gamma_y(m,v)$ denotes Gaussian probability density function in argument $y$, with a given mean $m$ and variance $v$.

### Bayesian calibration

*The usual steps of Bayesian calibration*

The complete form of a joint p.d.f. for calibration can be chosen from the theoretical models, and from knowledge and experience of experts in the field. However, it could be useful to sketch a common way of constructing a joint p.d.f. The usual steps are:

(1) The form of calibration curve $\hat{y} = F(x,\theta)$ is chosen; this specifies the dependence of the *expected* value of a measurement in a parametrized way, and assumes that the measured concentration is $x$.

(2) The distribution of the measurement errors is either guessed or estimated in a preliminary stage. The errors are assumed to be independent and zero mean (the systematic error is included in $F(x,\theta)$ mostly as an additive constant).

(3) The *forms* of the distribution of measurement errors, as well as that of the calibration curve, are assumed to be invariant during the whole calibration and measurement process. This property, of course, should be tested. Note that steps (1) to (3) specify the form of $p(Y|X,\beta)$.

(4) The distribution of sample preparation errors during control sample preparation is specified [adopting steps (1) to (3)]. This determines the form of $p(X|\beta)$ and, together with the form of $p(Y|X,\beta)$, the form of c.p.d.f. $p(Y,X|\beta)$ by the chain rule. For calibration the concentrations $X$ are mostly assumed to be error-free (see the Example).

(5) Expert knowledge is often reduced to uniform distribution on the range of possible parameter values. Then a preliminary experience accumulation stage (preliminary Bayesian calibration) is performed, resulting in the routinely used prior p.d.f.

$$p(\beta) = p(\beta|\text{ data in preliminary stage}).$$

The required c.p.d.f. $p(Y,X|\beta)$ is determined by steps (1)–(5). These steps should be applied iteratively in order to ensure computational feasibility of the resulting evaluations. For example those distributions with low dimensional sufficient statistics for $\beta$ estimation are preferable.

The identification of the parameters in the joint c.p.d.f. $p(X,Y|\beta)$ can be done following the standard Bayesian identification [7]. A Bayesian identification results in the c.p.d.f. of the unknown parameter $\beta$ conditioned on the past data $(X,Y)$, i.e. $p(\beta|Y,X)$. Having measured the data $(Y,X)$ and having given the prior distribution $p(\beta)$, the Bayesian estimate can be written in the form

$$p(\beta|Y,X) = \frac{p(Y,X|\beta)p(\beta)}{\int p(Y,X|\beta)p(\beta)\mathrm{d}\beta}. \qquad (10)$$

*Utilization of the calibration results*

The most important applications of the calibration results are as follows.

*Evaluation of unknown concentrations*

The aim of the measurement is to estimate the concentrations $(X)$ of unknown samples from their measured values $Y$. In the Bayesian framework the estimate of $X$ is given in terms of its c.p.d.f. $p(X|Y,Y_c,X_c)$, which is easily calculated from the c.p.d.f. (6) using the chain rule. For a single measurement $y$ of an unknown sample $x$, the resulting formula can be written explicitly in terms of the calibration result $p(\beta|Y_c,X_c)$, of the probabilistic model of the measurement process $p(y|x,\beta)$ and of the p.d.f. $p(x)$. The unknown sample population is then described as follows

$$p(x|Y_c,X_c) = Cp(x)\int p(y|x,\beta)p(\beta|Y_c,X_c)\mathrm{d}\beta \qquad (11)$$

where $C$ is a normalizing constant which is independent of $x$. This formula can be derived by applying elementary rules for p.d.f.s, following the restrictions setout in remark (5) of the section on the Bayesian view of the calibration problem.

*Remarks*

(1) The p.d.f. $p(x)$ which describes the population of unknown samples is not (seemingly) found in other approaches to calibration. It can be shown, however, that the constant $p(x)$ over the range of possible concentration values is implicitly used. Assuming that there is an equal chance of all values occurring $x$ then $p(x)$ = constant. Bayesian methodology allows non-trivial information to be incorporated and thus to improve the quality of the results.

(2) A suitable characteristic (for example expectation) given by the c.p.d.f. (11) can be taken as a (point) estimate of the unknown concentration $x$. At the same time, a description of the point estimate uncertainty [for example covariance matrix given by the c.p.d.f. $p(x|y,Y_c,X_c)$] is available.

(3) When calibrating, a balance has to be found between cost of the calibration and possible losses caused by imprecisely determined values of measured concentration. Clearly, any compromise must be based on information about the uncertainties of the treated quantities. The unique property of the Bayesian framework is the ability to supply this information, regardless of the amount of data available.

*Measurement control*

The quality of the calibration characterizing the measurement conditions is usually checked by standard hypothesis testing. The Bayesian calibration supports this stage because the information needed is contained in the c.p.d.fs supplied. Solving two typical problems explains this situation:

(a) The control samples are used to judge whether the parameters $\beta$ characterizing the measurement process are in a given acceptable range $D_\beta$. The probability of the hypotheses $H{:}\beta \varepsilon D_\beta$ (conditioned on results of measurements of control samples) can be directly computed from the c.p.d.f. given by equation (10)

$$p(H|Y,X) = \int_{D\beta} p(\beta|Y,X)\mathrm{d}\beta. \quad (12)$$

(b) Any calibration relies on the assumption that some characteristics do not change during the calibration and measurement phases. This assumption has to be tested in order to gain reliable analytical results. This problem can also be handled using the Bayesian approach as a special case of Bayesian model comparison [7].

The problem is as follows: there are two sets of data, say $D^i = (Y^i,X^i)$, $i = 1,2$ with a common functional form c.p.d.f. $p(Y^i, X^i|\beta)$, with prior p.d.f. $p(\beta)$ it is possible to formulate two hypotheses:

$H_1 : p(Y,X|\beta^1) = p(Y,X|\beta^2)$: parameters did not change;

$H_2 : p(Y,X|\beta^1) \neq p(Y,X|\beta^2)$: parameters changed.

The hypothesis $H$ has two possible values, $H_1$ and $H_2$ and becomes a random variable according to the Bayes methodology. Assigning prior probabilities, $p(H_i)$ to $H_i$, it is simple to determine the posterior probability of $H_1$ (and thus $H_2$) as

$$p(H_1|D^1,D^2) = \\ = \frac{1}{1 + \left(\frac{1}{p(H_1)} - 1\right)\frac{\int p(D^1|\beta)p(\beta)\mathrm{d}\beta \int p(D^2|\beta)p(\beta)\mathrm{d}\beta}{\int p(D^1|\beta)p(D^2|\beta)p(\beta)\mathrm{d}\beta}}$$

A detailed derivation and analysis of the above formula is out of the scope of this paper; it should be, however, noted that the prior probability $p(H_1)$ of the hypothesis $H_1$ is the only new item which has to be added.

It is natural that for linear models and *normally* distributed errors the evaluation deals with quantities similar to those in the ordinary $t$-test, but the use of $p(H|D^1,D^2)$ relies neither on asymptotic results nor on the significance level.

### Practical application of the Bayesian calibration

*Experimental conditions*

*Equipment and materials*

A Fractoven 4200 gas chromatograph with an HS-250 automatic head-space sampler (Carlo Erba, Milan, Italy) were used for the analyses. The samples were prepared by a Microlab M Diluter/Dispenser (Hamilton, Bonaduz, Switzerland). The chromatographic data were collected and evaluated by an HP-3354 B/C Laboratory Automation System (Hewlett-Packard, California, USA). The control samples were made of Alkohol-Standardlosung (Merck, Darmstadt, FR Germany). The stock solution of the internal standard was prepared in the authors' laboratory from 0·45 g/l propan-1-ol (chromatographic grade, Carlo Erba, Milan, Italy) and 200 g/l sodium sulphate (analytical-reagent grade, Merck, Darmstadt, FR Germany) diluted in doubly distilled water.

*Measurements*

0.2-ml volume of the control sample and 0·2 ml of the stock solution were measured into a 5-ml sampling vial and the vial was sealed. The samples in vials were equilibrated at 50 °C in the water-bath of the HS-250 for at least 20 min. Then a 0·8-ml gas sample was injected into the gas chromatograph by the HS-250 equipped with injection syringe 1001 LTSN (1 ml) (Hamilton, Bonaduz, Switzerland). The measurement was performed in sequences three times a day (at 9 a.m., 11 a.m. and 1 p.m.). In the sequence there were five samples of 0·5, 0·8, 1·0, 2·0 and 3·0 g/l ethanol content, all of which were measured in random order.

The *chromatographic conditions* were as follows: column, sililysed glass, 1·5 m long, 0·2 cm internal diameter filled with Carbopack C 800–100 mesh coated with 0·2% Carbowax, 1500; carrier gas, nitrogen, flow-rate 100 ml/min; detector, FID; injection septa, Tightsep 11 mm in diameter (Supelco, Bellefonte, Pennsylvania, USA). The *temperatures* used were: injection syringe, 95 °C; injector, 125 °C; detector, 125 °C; and column oven, 80 °C.

The quantitative evaluation was performed on the basis of the peak area and height ratio of ethanol and propan-1-ol.

*Models for calibration*

The models for calibration are derived using Bayesian calibration (see the section on the usual steps of Bayesian calibration). The necessary assumptions and models for head-space gas chromatography of liquid samples are as follows:

(1) The possible form of calibration curve is a line:

$$\hat{y} = F(x,\theta) = a + bx \quad (13)$$

where $a$ and $b$ are unknown parameters forming the vector $\theta$.

(2) The measurement error of the $i$th measurement $\zeta_i$ is assumed to have a log-normal distribution with concentration dependent variance, i.e. the measured values are

$$y_i = (a + bx_i)\zeta_i \quad (14)$$

or equivalently

$$\log(y_i) = \log(a + bx_i) + \varepsilon_i \quad (15)$$

where $\varepsilon_i$ has Gaussian distribution with zero mean and unknown variance $s>0$. The measurement errors are assumed to have a genuinely random nature, i.e. the components $\zeta_1, \zeta_2, \ldots$ are identically distributed and mutually independent.

The advantage of this model (14) includes (a) the higher the expected value of observations then the higher the variance is assigned to them by log-normal distribution. This feature matches all practical experience with the described case; (b) log-normal distribution models the observations with outliers so the calibration relying on it will be sound with respect to outlying data; (c) a linearized form of the (probabilistic) model (15) is easily tractable computationally [7].

(3) If the linearized form of (15) is time-invariant then the (linearized) calibration model is specified. For its description we use

$$z_i = \log\left(\frac{y_i}{x_i}\right) \tag{16}$$

$$u_i = \frac{1}{x_i} \tag{17}$$

$$A = \log(b) \tag{18}$$

$$B = \frac{a}{b} \tag{19}$$

Then

$$p(Z|U,\beta) = C \prod_{i=1}^{N} \Gamma_z(A + Bu_i, s) \tag{20}$$

$$\text{with } \beta = (A,B,s). \tag{21}$$

(4) The concentrations of the control samples are assumed to be error-free, thus

$$p(X|\beta) = \delta(x - X) \tag{22}$$

and the calibration results can be computed according to equations (7) and (10).

(5) The prior distribution is chosen to be in the self-reproducing Gauss-Wishart form [7] characterized by *a priori* independent values of $\beta = (A,B,s)$ with prior expectation $(0,0,10^{-2})$. This choice is equivalent to the assumption that there will be zero offset and unit slope in (13) and measurement errors of the order $10^{-1}$. The variances were chosen to assign about 95% of prior probability to the assumption that the offset is in the range (−0·5, 0·5) and the slope is in the range (0·8, 1·2).

*Calibration results*

Typical sequences of measured data are shown in tables 1 and 2. Other series with outliers are shown in tables 3 and 4.

*Table 1. Typical data of peak area ratio obtained for three different sequences of control samples.*

| Time of measurement | Ethanol concentration/gl$^{-1}$ | | | | |
|---|---|---|---|---|---|
| | 0·5 | 0·8 | 1·0 | 2·0 | 3·0 |
| 9 a.m. | 0·4725 | 0·765 | 0·9754 | 2·0316 | 3·0588 |
| 11 a.m. | 0·4621 | 0·7701 | 0·9709 | 2·0384 | 3·0229 |
| 1 p.m. | 0·4611 | 0·7608 | 0·9848 | 2·0022 | 3·0751 |

*Table 2. Typical data of peak height ratio obtained for three different sequences of control samples.*

| Time of measurement | Ethanol concentration/gl$^{-1}$ | | | | |
|---|---|---|---|---|---|
| | 0·5 | 0·8 | 1·0 | 2·0 | 3·0 |
| 9 a.m. | 1·0649 | 1·7014 | 2·1633 | 4·4546 | 6·6793 |
| 11 a.m. | 1·0376 | 1·7039 | 2·1425 | 4·5068 | 6·6214 |
| 1 p.m. | 1·029 | 1·7092 | 2·1722 | 4·4374 | 6·7309 |

*Table 3. Typical data of peak area ratio obtained for three different sequences of control samples with outliers.*

| Time of measurement | Ethanol concentration/gl$^{-1}$ | | | | |
|---|---|---|---|---|---|
| | 0·5 | 0·8 | 1·0 | 2·0 | 3·0 |
| 9 a.m. | 0·6112 | 0·745 | 1·0964 | 1·9914 | 3·1132 |
| 11 a.m. | 0·5888 | 0·7613 | 1·0026 | 2·0573 | 3·0901 |
| 1 p.m. | 0·5162 | 0·9889 | 0·9707 | 2·0529 | 3·1411 |

*Table 4. Typical data of peak height ratio obtained for three different sequences of control samples with outliers.*

| Time of measurement | Ethanol concentration/gl$^{-1}$ | | | | |
|---|---|---|---|---|---|
| | 0·5 | 0·8 | 1·0 | 2·0 | 3·0 |
| 9 a.m. | 1·2994 | 1·6552 | 2·3774 | 4·4056 | 6·8293 |
| 11 a.m. | 1·3075 | 1·7033 | 2·2268 | 4·5161 | 6·7518 |
| 1 p.m. | 1·1356 | 2·1753 | 2·156 | 4·5522 | 6·6182 |

*Table 5. The estimated parameters.*

| Case No. | $\hat{s}$ (variance est.) | $\hat{b}$ (slope est.) | $\hat{a}$ (offset est.) | Remark |
|---|---|---|---|---|
| 1 | $5{\cdot}806\ 10^{-4}$ | 1·021 | $-4{\cdot}483\ 10^{-2}$ | Peak area ratio, no outliers |
| 2 | $2{\cdot}180\ 10^{-2}$ | 1·929 | $1{\cdot}370\ 10^{-1}$ | Peak height ratio, no outliers |
| 3 | $3{\cdot}866\ 10^{-3}$ | 0·998 | $4{\cdot}862\ 10^{-2}$ | Peak area ratio with outliers |
| 4 | $2{\cdot}330\ 10^{-2}$ | 1·897 | $2{\cdot}859\ 10^{-1}$ | Peak height ratio with outliers |

The estimated parameters for the four different cases are collected in table 5.

Comparison of the results of corresponding cases 1, 3 and 2, 4 in table 5 gives experimental support for the robustness claimed: the estimates in corrupted and uncorrupted cases do not significantly differ.

Figure 1 illustrates the results of the Bayesian calibration (for case 2). On the c.p.d.f. $p(y|x, Y_c, X_c)$ drawn it can be seen that the probability of observing various values of $y$ is not symmetrical and has a heavy tail which reflects the presence of outliers; and that these variance increases with increasing values of the measured concentration.

It is assumed that all values of concentration are equally probable in the population of unknown samples, the c.p.d.f. is proportional to $p(x|y, Y_c, X_c)$ [cf. (11)]. Thus taking its section at a fixed measured value $y$ the probability of concentrations which could cause this value can be seen.

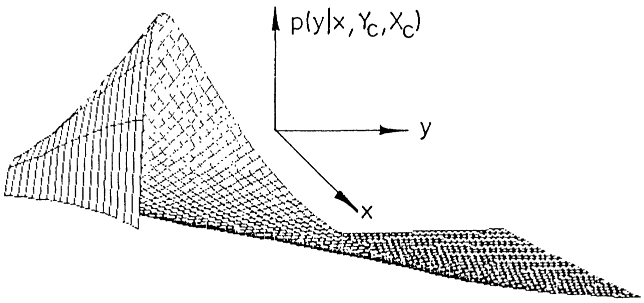

*Figure 1. Conditional probability density function* p(y|x,$Y_c$,$X_c$) *of possible measured data* y *for various concentrations* x. (*The calibration data* $Y_c$,$X_c$ *containing outliers are shown in table 2*).

### Conclusion

The Bayesian calibration method has been described with emphasis on practical aspects of its application. In order to encourage its wider use, the basic steps of Bayesian calibration are reviewed and possible applications of the results are given.

The calibration related to head-space gas chromatographic data was described to show the advantages of Bayesian calibration. The linear calibration case has been treated with log-normally distributed measurement noise. This treatment of noise stresses the (often overlooked) importance of modelling random constituents in any problem.

The authors hope that the paper has demonstrated that Bayesian calibration is especially advantageous when used for measurement control because information about uncertainty in the results after calibration is supplied.

### Acknowledgements

Dr Piroska Bujtás, performed the head-space gas chromatographic measurements and the authors are grateful for her help.

### References

1. Hilton, J., Talling, I. B., Clark, R. T., Calibration in routine analytical chemistry. *Laboratory Practice,* **10** (1982), 990–992.
2. Kateman, G., New directions in calibration. *Trends in Analytical Chemistry,* **2** (1983), IX-X.
3. Hunter, W. G. and Lamboy, W. F., A Bayesian analysis of the linear calibration problem (with discussion). *Technometrics,* **22** (1981), 323–350.
4. Hangos, K. M. and Leisztner, L., The systematic error caused by random errors through data reduction. *Journal of Automatic Chemistry,* **9** (1987), 25–29.
5. Hangos, K. M., Nagy, J. L., Leisztner, L., Automatic detection of the autocorrelation-type measurement error component. *Journal of Automatic Chemistry,* **8** (1986), 23–27.
6. Gnedenko, B. V., *Theory of Probability.* (Fizmatgiz, Moscow, 1973) (2nd printing).
7. Peterka, V., Bayesian approach to system identification. In P. Eykhoff (ed.): *Trends and Progress in System Identification,* (Pergamon Press, Oxford, 1981).
8. Kárný, M., Quantification of global knowledge about the controlled system. *Kybernetika,* **20** (1984), 376–385.
9. Kárný, M., and Hangos, K. M., Decision-theoretical formulation of calibration problems. *Journal of Automatic Chemistry,* **11** (1989), 70–75.
10. Frank, J. E., Beyond linear least squares regression. *Trends in Analytical Chemistry,* **6** (1987), 271–275.
11. Unadkat, J. D., Beal, S. L., Sheiner, L. B., Bayesian Calibration. *Analytica Chemica Acta,* **181** (1986), 26–36.
12. Clancey, V. J., Statistical Methods in Chemical Analyses. *Nature,* **159** (1947), 339–340.

